# Characterizing the malignancy and drug resistance of cancer cells from their membrane resealing response

**DOI:** 10.1038/srep26692

**Published:** 2016-05-26

**Authors:** T. H. Hui, Z. L. Zhou, H. W. Fong, Roger K. C. Ngan, T. Y. Lee, Joseph S. K. Au, A. H. W. Ngan, Timothy T. C. Yip, Y. Lin

**Affiliations:** 1 Department of Mechanical Engineering, The University of Hong Kong, Hong Kong SAR, China; 2 HKU-Shenzhen Institute of Research and Innovation (HKU-SIRI), Shenzhen, Guangdong, China; 3 Department of Clinical Oncology, Queen Elizabeth Hospital, Hong Kong SAR, China

## Abstract

In this report, we showed that two tumor cell characteristics, namely the malignancy and drug-resistance status can be evaluated by their membrane resealing response. Specifically, membrane pores in a number of pairs of cancer and normal cell lines originated from nasopharynx, lung and intestine were introduced by nano-mechanical puncturing. Interestingly, such nanometer-sized holes in tumor cells can reseal ~2–3 times faster than those in the corresponding normal cells. Furthermore, the membrane resealing time in cancer cell lines exhibiting resistance to several leading chemotherapeutic drugs was also found to be substantially shorter than that in their drug-sensitive counterparts, demonstrating the potential of using this quantity as a novel marker for future cancer diagnosis and drug resistance detection. Finally, a simple model was proposed to explain the observed resealing dynamics of cells which suggested that the distinct response exhibited by normal, tumor and drug resistant cells is likely due to the different tension levels in their lipid membranes, a conclusion that is also supported by direct cortical tension measurement.

Currently, diagnosis of cancers largely relies on biopsy^1^,^2^, a method inevitably involving analyzing huge amount of biological information (like the wide spectrum of morphological features of cells) and then making, to certain extent, “subjective” interpretations which unfortunately can lead to incomplete or misleading differential diagnosis^3^. In addition, more accurate molecular classification of cancers, such as drug resistance identification, often requires long time colony culturing and screening^4^,^5^ (taking days or even weeks to complete) which may prevent the patients from receiving immediate treatment that could be crucial. As such, finding reliable and fast ways to differentiate cancer cells from normal ones, as well as among themselves, has always been an area of great interest. For example, significant progress has been made in recent years in utilizing different nanoparticles for cancer detection purposes^6^,^7^, although their cytotoxicity remains to be a concern^8^,^9^.

Interestingly, accumulating evidence has shown that the physical properties of cells are intimately related to their pathological state^10^,^11^,^12^,^13^,^14^,^15^,^16^. For instance, it was found that tumor cells are often softer than their normal counterparts^17^ and their elastic moduli correlate with their metastatic potentials^17^,^18^,^19^. The rigidity of bacteria has also been reported to be significantly influenced by their drug resistance status^20^. However, measuring the stiffness of cells demands precise control over the position of the probe that deforms the cell and/or the force exerted by it, making it rather difficult to achieve a high throughput. In addition, it also appears to us that the uniqueness and robustness of using stiffness as a marker for cancer detection has not been rigorously established.

In this study, we used a mechanical puncturing approach (see Supplementary information A) to create nanometer-sized pores on the lipid bilayer in tumor and non-tumor cell lines from three different human organs, namely, nasopharynx, lung and intestine (culture protocols and cell lines are given in Supplementary information B), and then investigated their spontaneous resealing. Interestingly, it was found that the tumor status of the cells can be efficiently distinguished by how fast the membrane pores are resealed. Furthermore, we also showed that the resealing behavior of tumor cells exhibits a strong correlation with their anti-cancer drug resistance (refer to Supplementary Information C for details). Finally, through theoretical modeling and direct measurement, we demonstrated that the distinct resealing response observed here is likely due to the different tension levels in the lipid membrane of normal, drug-sensitive and drug-resistant cancer cells.

## Results

### Spontaneous resealing of nano-sized membrane pores

**Figure 1:**
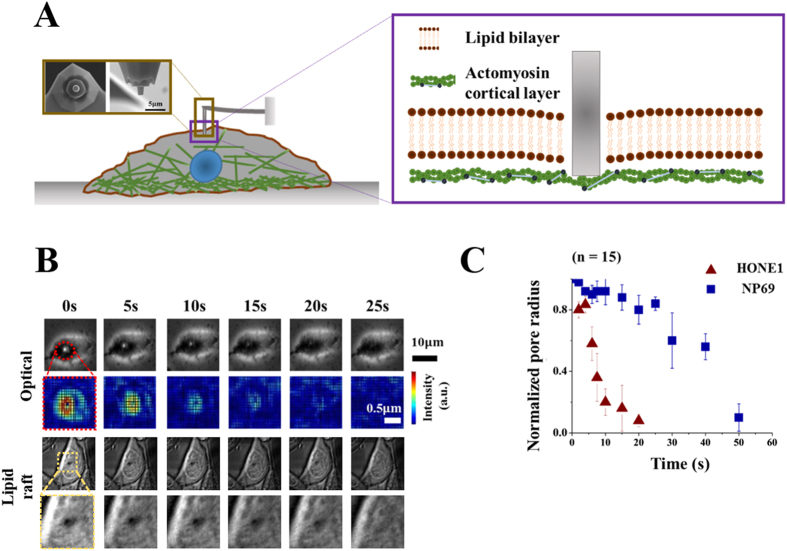
Nanomechanical puncture test. (**A**) Schematics of the nano-mechanical puncturing test where a flat-end cylindrical indenter is used (with actual images given in the inset) to penetrate and create a pore in the lipid bilayer membrane. (**B**) Representative optical (top) and lipid raft (bottom) images showing the resealing dynamics of a membrane pore in HONE1 cell. Amplified plots (after smoothing by a cstomized computer program in the optical case) are also given under the original ones. Note that, the black region in the lipid raft image has essentially zero lipid intensity indicating that the membrane was ruptured there. (**C**) Temporal evolution of the radii of membrane pores, normalized by the initial value 0.5 μm (i.e. the radius of the indenter used), in HONE1 and NP69 cells. Error bar represents the standard deviation from 15 independent measurements for each cell type. A statistical confidence level of no less than 97% by t-test has been achieved in (**C**).

Membrane pores in the nasopharyngeal carcinoma (NPC) cell line, HONE1 were created by a 500 nm -radius AFM indenter (Fig. 1A). Spontaneous resealing of the holes was monitored by taking snap shot images at a frame rate of 0.1 s^−1^ after removal of the indenter. Figure 1B shows that a membrane pore in HONE1 will typically disappear in ~20 seconds. To confirm whether the membrane was ruptured by the indenter, we have labeled the membrane fluorescently with lipid raft. As shown in the bottom panel of Fig. 1B, the lipid intensity in the punctured hole was essentially zero initially, manifested as a black region, demonstrating that the membrane is indeed ruptured. Interestingly, the resealing time for an immortalized normal nasopharyngeal cell line, NP69, was 250% longer (approximately 50 seconds, refer to Fig. 1C).

**Figure 2:**
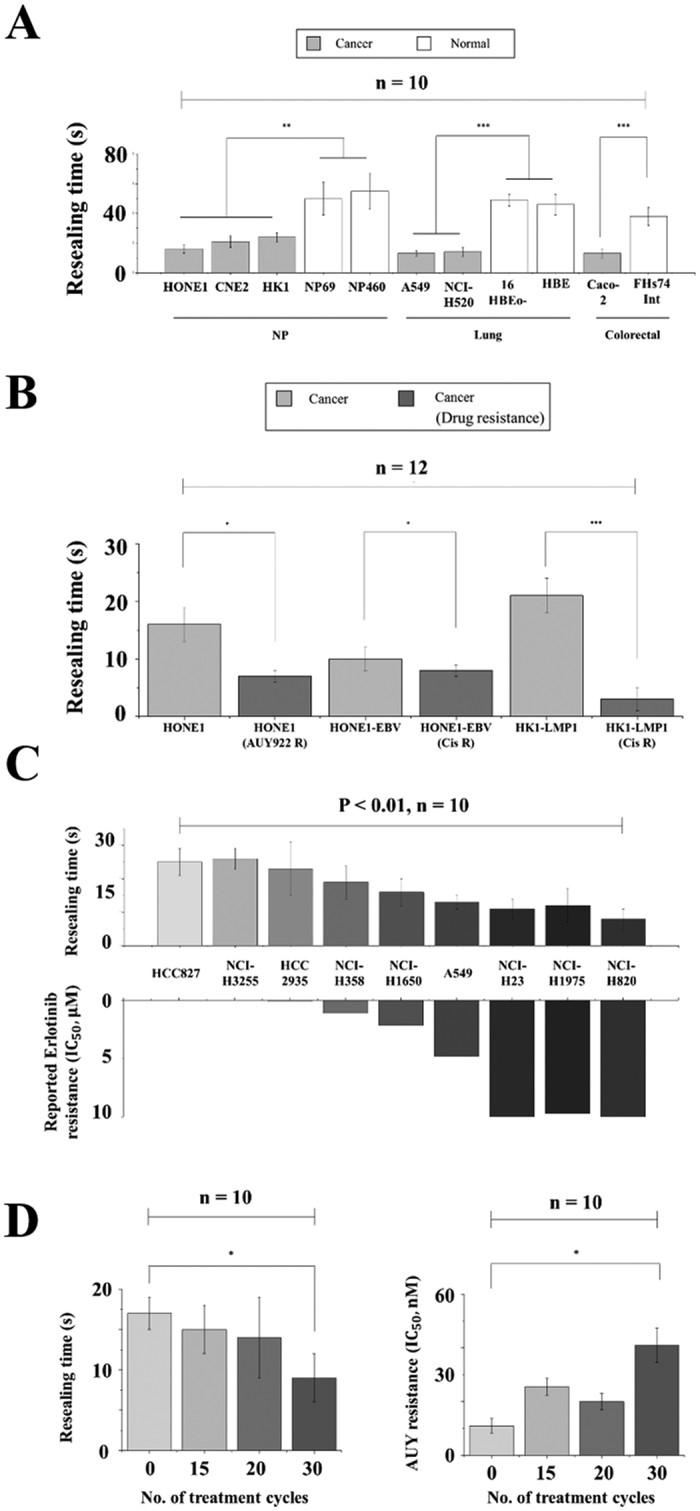
Resealing time comparison among cell lines. (**A**) Comparison between the measured resealing time of 1 μm membrane pores in different cancer and corresponding normal cell lines. (**B**) Bar plots of the resealing time of nasopharyngeal drug-resistant cell lines (HONE1-AUY R, HONE1-EBV Cis R and HK1-LMP1 Cis R) along with their parental cell lines (HONE1, HONE1-EBV, and HK1-LMP1) that are drug-sensitive. (**C**) Bar plots of the resealing time of lung cancer cells with different reported Erlotinib resistance (characterized by the critical Erlotinib concentration required to achieve 50% growth inhibition, i.e.IC_50_). (**D**) The resealing time of HONE1 cell line in different phases of AUY922 treatment cycles where the drug-resisting capability of cells is described by IC_50_ of AUY922, similar to that in (**C**). Asterisks here denote a statistically significant difference by t-test (*P < 0.05, **P < 0.01, ***P < 0.001) between paired samples and n stands for the number of independent trials. Results shown in (**A**–**C**) all have a statistical significance of P < 0.01 by one sample t-test while P < 0.05 in (D).

To examine whether such a huge difference is a general feature between cancer and normal cells, we systematically examined the resealing response in cell lines derived from three anatomical positions, namely, nasopharynx (NPC cell lines - HONE1, CNE2 and HK1 versus immortalized normal NP cell lines – NP69 & NP460), lung (lung cancer cell lines - A549 & NCI-H520 versus immortalized normal lung cell lines – HBE & 16HBEo-) and intestine (intestinal cancer cell line - Caco-2 versus intestinal normal cell line – FHs74Int). Interestingly, cancer cells were found to always reseal 200% to 300% times faster than the corresponding normal ones irrespective of the organs from which they were established (Fig. 2A).

More surprisingly, the resealing behavior of cancer cells also appears to be influenced by their drug-resistance status. Specifically, two pairs of cancer cell lines with and without resistance to Cisplatin (i.e. HONE1-EBV CisR & HK1-LMP1 CisR versus HONE1-EBV & HK1-LMP1 respectively), a drug widely used in treating head, neck and ovarian cancer patients, and one pair of NPC cell lines with and without resistance to AUY922, a drug targeting heat shock protein-90 (HSP90), were tested. All drug resistant cell lines exhibit a much shortened resealing time as compared to their non-resistant counterparts (Fig. 2B). Another commonly used drug in treating non-small cell lung cancer (NSCLC) patients with epidermal growth factor receptor (EGFR) gene mutation is Erlotinib. Despite its efficacy, resistance can develop after prolonged usage of Erlotinib resulting in cancer relapse in many patients. To test whether the membrane resealing time is correlated with the resistance to Erlotinib, we performed the same membrane resealing experiments in nine different well-established lung cancer cell lines (HCC827, NCI-H3255, HCC2935, NCI-H358, NCI-H1650, A549, NCI-H23, NCI-H1975& NCI-H820). Indeed, our results showed that how fast membrane pores in these cells can reseal correlated well in a reversed stepwise fashion with their reported Erlotinib resistance^21^,^22^, refer to Fig. 2C.

Finally, to further confirm the reverse trend between the resealing time and drug-resistant characteristic, we developed a series of HONE1 sub-lines with gradual increase of resistance to AUY922 by pulsing HONE1 with the drug repeatedly for 6 months (Fig. 2D). Again, HONE1 subclones with increasing levels of AUY922 resistance exhibited a gradual decrease in the resealing time (Fig. 2D).

### A theoretical model for pore resealing

**Figure 3:**
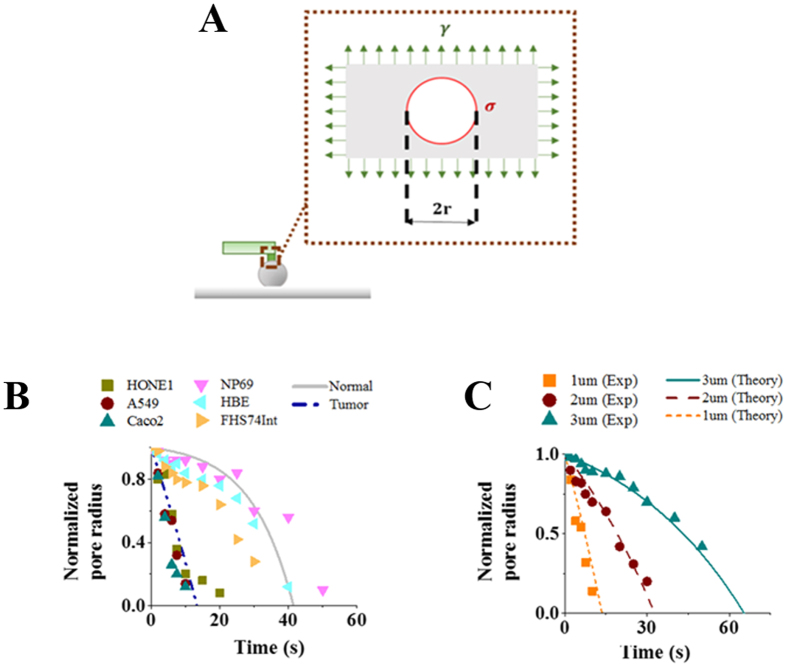
Puncture resealing model. (**A**) Schematic diagram illustrating the resealing of a membrane pore governed by its edge energy density σ and bi-layer tension *γ*. (**B**) Temporal evolution of the normalized pore radius. Experimental data are represented by markers while predictions from Eq. (2) are shown by lines where *γ* is chosen as 8 pN/μm and 34.5 pN/μm for tumor and normal cells, respectively. Other parameters adopted here are listed in Supplementary Information Table 2. (**C**) Resealing response of membrane holes with different initial diameters (1, 2 or 3 μm). Markers here correspond to measurement data on A549 cells while lines represent predictions from Eq. (2) under a fixed *γ* of 8 pN/μm. Experimental results shown in (**B**,**C**) were based on measurements on 15 live cells with *P* < 0.03 by one-sample t-test.

To better understand the distinct resealing response observed here, recall that, from a thermodynamics point of view, the free energy of a membrane pore, with radius r, can be expressed as^23^,^24^


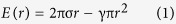


where γ is the lipid bilayer tension while σ is the edge energy (or line tension) associated with the peripheral of the pore (Fig. 3A). Physically, σ originates from the fact that the hydrophobic tails of lipid molecules are exposed to water at the pore edge leading to an increase in the free energy. Once E is known, the size evolution of the pore can then be described by 
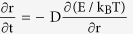
 where k_B_T is the thermal energy and D is a parameter representing how fast the pore edge can move. Following this reasoning, the pore radius as a function of time is predicted to be,


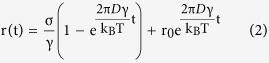


with r_0_ being the initial radius of the hole (i.e. the radius of the cylindrical probe used to puncture the cell wall). Given that the structure and diffusivity of lipid molecules in different cells are unlikely to vary significantly, the values of σ and D should be more or less the same across cell types (the adopted values of these two parameters, compared favorably to those reported in the literature, are listed in Supplementary Table 2). As such, the key factor affecting the resealing dynamics concerned here is γ, noticing that r will decrease monotonically with respect to time as long as r_0_ < σ/γ according to Eq. (2).

Interestingly, within this theoretical framework, the observed big difference in the resealing time of tumor and normal cells can be well explained by assuming that the tension level in cancer cells is much lower than that in the corresponding normal ones. In particular, choosing *γ* = 8 pN/μm, prediction from Eq. (2) matches well the observed size evolution of membrane pores, with an initial size of 500 nm in radius, in different cancerous cell lines such as HONE1, A549 and Caco-2. On the other hand, experimental data from normal cell lines such as NP69, HBE and FHs74Int can be well-fitted by Eq. (2) if the bilayer tension is taken to be 34.5 pN/μm (Fig. 3B,C). We also compared predictions from Eq. (2) with observations regarding how membrane pores with different initial diameters (1, 2 or 3 μm) evolve in A549 cell line. As shown in Fig. 3C, excellent agreement between theory and experiment has been achieved for a fixed γ at 8 pN/μm.

**Figure 4:**
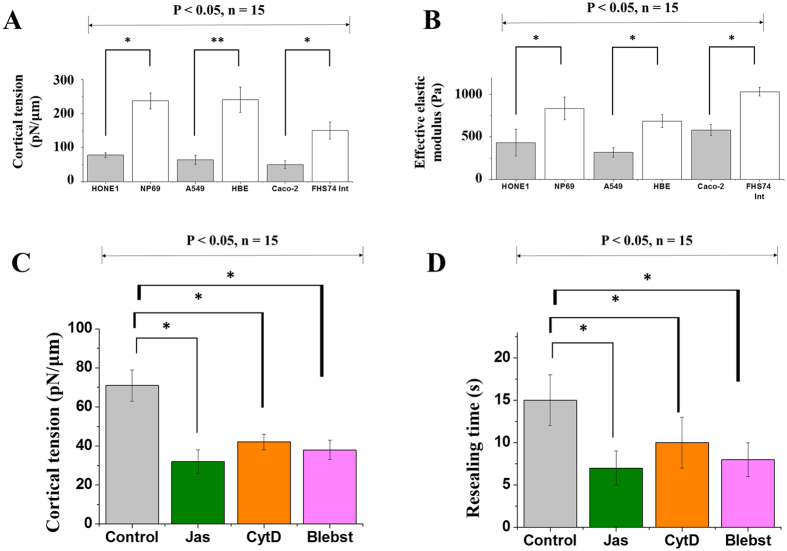
Correlation between resealing time and cortical tension. (**A**) Measured cortical tension in different tumor and corresponding normal cells. (**B**) Effective elastic moduli of the cells listed in (A). (**C**) Measured cortical tension in Jas-, CytD- or Blebst-treated A549 cells. (**D**) Measured membrane resealing time in Jas-, CytD- or Blebst-treated A549 cells. Asterisks here have the same meanings as those defined in Fig. 2. Results shown here were based on measurements on 15 cells (in each case) and a statistical confidence level of no less than 95% by t-test has been achieved.

To further confirm notion that the low resealing speed in normal cells, as compared to cancer ones, is caused by the high tension levels in their lipid membranes, we have conducted two sets of additional experiments. In the first one, the cortical tension (γ_m_) of cells was measured via nano-indentation (refer to Supplementary Information D). Indeed, it was found that this quantity in tumor cells is much lower than that in their normal counterparts (see Fig. 4A). Notice that the cortical tension is different from the lipid bilayer tension appeared in the resealing model described above. Actually, γ_m_ (generated by the passive deformation of the actin cortex as well as the active contraction of myosin motors inside) is expected to be much larger than γ^25^. Although we cannot measure the bilayer tension directly, it is reasonable to believe that a positive correlation between γ_m_ and γ exists, that is a higher level of γ_m_ will be accompanied by a higher level of γ and vice versa. Secondly, we monitored the resealing response of A549 cells treated with Jasplakinolide (Jas), Cytochalasin D (CytD) and Blebbistatin (Blebst). Jas and CytD are known to disrupt the actomyosin assembly in the cell cortex while the contractility of myosin will be inhibited by Blebst (see Supplementary Information E for details). As expected, a marked reduction in the resealing time, coupled with a decrease in the cortical tension, was observed in cells treated with Jas, CytD or Blebst (Fig. 4C,D). The resealing response of A549 cells undergoing hyperosmotic treatment was also examined (refer to Supplementary Information F). Basically, it was found that a higher increase in the medium osmolarity will lead to a larger shrinkage of cells as well as a faster resealing response (Supplementary Figure 4) which is consistent with our model predictions.

Lastly, to explore possible correlation between the stiffness and membrane resealing behavior of cells, the effective elastic moduli of different cancer (HONE1, A549 and Caco-2) and corresponding normal (i.e. NP69, HBE and FHS74Int) cell lines were measured by rate-jump indentation (see Supplementary Information G). As shown in Fig. 4B, tumor cells all appear to be softer than their normal counterparts, in agreement with previous studies^17^.

## Discussion

In summary, here we demonstrated the potential of using membrane resealing time as a novel marker in differentiating cancer cells from their normal counterparts. Specifically, it was shown that the membrane pores in various cancer cell lines derived from three anatomical positions, (namely, nasopharynx, lung and intestine) all reseal 200% to 300% faster than those in the corresponding normal ones. More interestingly, we found that there is a strong correlation between the resealing response of tumor cells and their capability to resist anti-cancer drugs with a clearly shortened resealing time in different cancer cells with resistance against commonly used drugs including Cisplatin, AUY922 and Erlotinib. Given that current drug sensitivity tests, no matter by colony, dye exclusion assays or other biochemical means, often require days to even weeks to complete and the results generated are usually far from accurate enough for clinical decision making. Our approach may provide an alternative for achieving rapid drug-resistance identification in the future.

Our analysis and cortical tension measurement suggest that the distinct resealing response is likely due to the different tension levels in the lipid membrane of cells. Actually, previous studied have indeed found that several cancer (including leukemia and cervical carcinoma) cells have a lower cortical tension (or elasticity)^11^,^26^ compared to their normal counterparts that possess a better differentiated cytoskeleton^26^. In addition, it has also been reported that the membrane can reseal faster in cells with a disrupted^27^ or rearranged^28^,^29^ (in the phase of mitosis) cytoskeletal structure, in consistent with our observations that cells treated with Jas, CytD, Blebst or hyperosmotic medium all exhibit a significantly reduced membrane resealing time. It is natural to postulate that the rapid resealing ability might provide a survival advantage for tumor cells by reducing the risk associated with membrane damages. If that is so, can we manipulate the tension level of cancer cells to make them more vulnerable or reduce their drug resistance? Experiments will be planned to examine these questions by first looking at the phenomenon in a larger variety of cancer cell lines derived from other organs such as breast, ovary, liver, skin, prostate, kidney, gastric gland and brain. In addition, since techniques like electroporation^30^,^31^ and sonoporation^32^,^33^ have all been well-established allowing us to create membrane holes in hundreds or thousands of cells simultaneously, it is conceivable that findings here could lead to high throughput cancer screening strategies in the future. Investigation along this line is underway.

## Materials and Methods

### Nano-mechanical puncturing

The cell puncturing experiments were conducted in a JPK NanoWizard^®^ II AFM (JPK Instrumental). A probe with a flat ended silicon nitride tip (Microlevers, Veeco) of diameter of 1, 2 or 3 μm, and a cantilever spring constant of 0.035 N/m, was used. The AFM tip was first made to indent into the cell at a speed of 0.2 μm/s until an indentation depth of 0.4 μm was reached. This was followed by holding for 30 s, and then retraction at a speed of 0.1 μm/s. Size evolution of the hole in the plasma membrane was captured by time-lapse images, taken at a rate of 10 frames/sec, and then quantitatively analyzed by a MATLAB program (see Supplementary Information A and Supplementary Figure 1 for details). All tests were conducted at 25 °C and within 1 hour after the cells were removed from the incubator.

### Cell lines and culturing protocols

The histology and source of all cell lines used in this study are provided in Supplementary Information B. Cells were maintained at 37 °C and 5% CO_2_humidified atmosphere. In addition, a total concentration of 2 × 10^5^ cells/mL was incubated in confocal dishes without serum/growth factor for 24 hours to achieve G0 phase synchronization prior to the actual test. Further details of the culturing protocols can be found in the Supplementary Information B.

### Fluorescent staining of lipid by lipid raft

To visualize the membrane dynamics, we stained the lipid domains of HONE1 cells by lipid raft. The labeling was performed by using the Vybrant Alexa-Fluor-488 Lipid Raft labeling kit, following the manufacturer’s protocol (Invitrogen). Specifically, HONE1 cells were washed with ice-cold PBS three times and incubated with fluorescent CT-B. Then anti-CT-B antibody was added to crosslink the CT-B in the lipid rafts into patches readily to be visualized by fluorescence microscopy. Cells were kept at 37 °C with 5% CO_2_ supply during the experiment.

### Drugs and protocols for developing drug-resistance

Supply of HSP-90 inhibitor drug, AUY922 was obtained from Novartis Pharmaceuticals (HK) Ltd. The NPC sub-line (HONE1-AUY922-R) which is resistant to AUY922 was established by pulsing HONE1 cell line with 30 nM AUY922 repeatedly every two to three days for six months until drug resistance is developed. The cisplatin-resistant cell lines (HK1-LMP1 CisR and HONE1-EBV CisR) were provided by Professor Brigette Ma & Dr. Eric Wong from the Department of Clinical Oncology, Chinese University of Hong Kong. The whole series of lung cancer cell lines with and without resistance to the anti-EGFR targeted drug, Erlotinib were acquired from ATCC. For other details of cell lines and drug resistance nature, please refer to Supplementary Information B and C.

### Cortical tension measurement

The same protocol used in the mechanical puncture tests, without the retraction stage at the end, was adopted here. In particular, the contact force between the cell and the cylindrical probe during the indenting and subsequent holding stages was recorded which, in conjunction with a simple model, enabled the so-called cortical tension of cells to be estimated (refer to Supplementary Information D for details). In our experiments, each cell was indented five times at different locations.

## Additional Information

**How to cite this article**: Hui, T. H. *et al.* Characterizing the malignancy and drug resistance of cancer cells from their membrane resealing response. *Sci. Rep.* **6**, 26692; doi: 10.1038/srep26692 (2016).

## Supplementary Material

Supplementary Information
