# Supplementary material for: Characterizing the malignancy and drug resistance of cancer cells from their membrane resealing response
Source: Sci Rep. 2016 May 26;6:26692. doi: 10.1038/srep26692 (PMC4880901; doi:10.1038/srep26692)
Supplement: Supplementary Information [file srep26692-s1.pdf]

TITLE: Characterizing the malignancy and drug resistance of cancer cells from their membrane resealing response

AUTHORS: T.H. Hui[1,2], Z.L. Zhou[1], H.W. Fong[3], Roger K.C. Ngan[3], T.Y. Lee[3], Joseph S.K. Au[3], A.H.W. Ngan[1], Timothy T.C. Yip[3,*] and Y. Lin[1,2*]

* Corresponding authors. E-mail: ylin@hku.hk, yiptc@ha.org.hk

AFFILIATION:

1 Department of Mechanical Engineering, The University of Hong Kong, Hong Kong SAR, China

2 HKU-Shenzhen Institute of Research and Innovation (HKU-SIRI), Shenzhen, Guangdong, China

3 Department of Clinical Oncology, Queen Elizabeth Hospital, Hong Kong SAR, China

A. Nano-mechanical puncture experiment.

The cell puncturing experiments were conducted in a JPK NanoWizard® II AFM (JPK Instrumental). A probe with a flat ended silicon nitride tip (Microlevers, Veeco) of diameter of 1, 2 or 3μm, and a cantilever spring constant of 0.035 N/m, was used. The AFM tip was first made to indent into the cell at a speed of $0.2\,\mu m/s$ until an indentation depth of 0.4μm was reached. This was followed by holding for 30s, and then retraction at a speed of $0.1\,\mu m/s$. Size evolution of the hole in the plasma membrane was captured by time-lapse images, taken at a rate of 10 frames/sec, and then quantitatively analyzed by a MATLAB program (Supplementary Fig. 1A and 1B for details). All tests were conducted at 25℃ and within 1 hour after the cells were removed from the incubator.

To quantitatively determine how membrane pores reseal, live cell video was loaded to a MATLAB program where image sequences were transformed into grayscale. The intensity of each pixel in the image was measured and the entire picture was then smoothened by an average as well as a low-pass filter, followed by pixel interpolation (see Supplementary Fig. 1). Since that the pore region was displayed with a different intensity (manifested as the white spot in the image as shown in Fig. 1B) from the rest of the membrane, its actual size at each time point could be evaluated by simple pixel counting in our program. The so-called resealing time was then determined by identifying the time point when the pore radius vanishes.

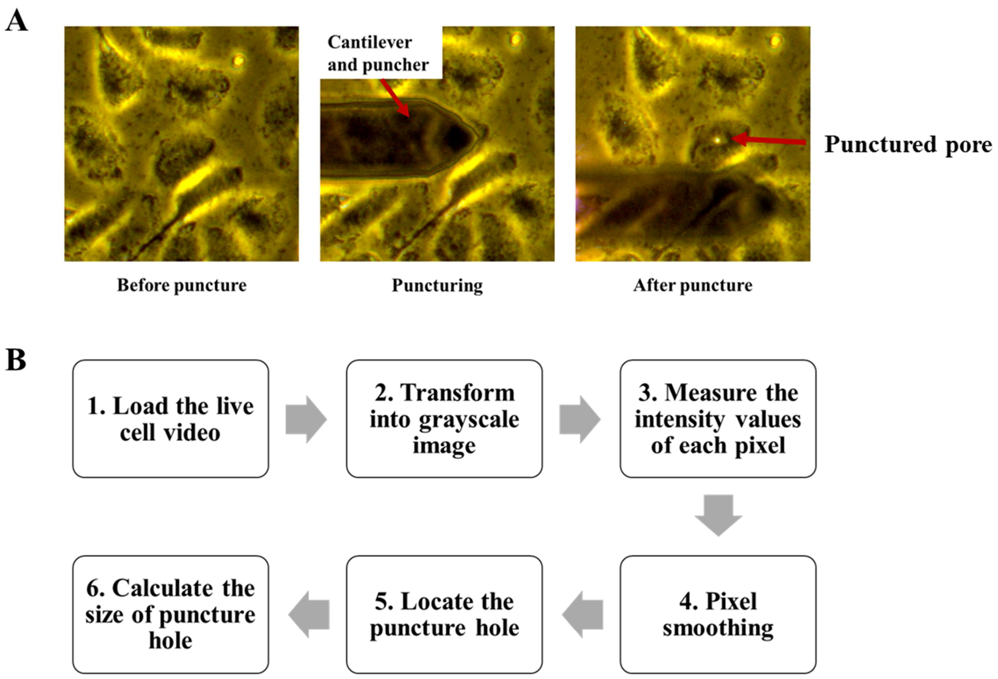

Supplementary Figure 1 **A** - Micrographs showing the appearance of a membrane pore in HONE1 cell after mechanical puncture. **B** - Flow chart of the computer program in analyzing the size evolution of membrane pores in the cell membrane puncture experiment.

Interestingly, it was found that the resealing time actually depends on the indentation depth to certain extent as shown in Fig. S2. However, such dependence disappears once the indentation depth is beyond 0.4 µm. For this reason, all our tests were conducted at this fixed penetration depth.

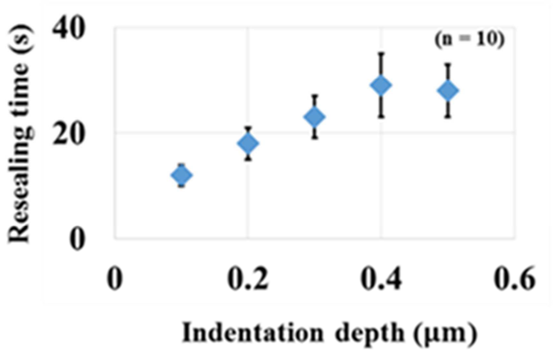

Supplementary Figure 2 Influence of the indentation depth on the membrane resealing time. Error bar represents the standard deviation of 10 independent measurements.

B. Cell lines used and culture conditions.

The two nasopharyngeal carcinoma (NPC) cell lines (HONE1 and CNE2) plus the two immortalized normal (or non-tumor) nasopharyngeal (NP) cell lines (NP69 & NP460) were provided by Professor George S.W. Tsao from the Anatomy Department, University of Hong Kong. The NPC cell line (HK1) was established by the late Professor Dolly Huang in Queen Elizabeth Hospital. The NPC sub-line (HONE1-AUY922-R) which is resistant to an inhibitor drug, AUY922 targeted to heat shock protein-90 (HSP90) was established in Queen Elizabeth Hospital by pulsing HONE1 cell line with 30 nM AUY922 repeatedly every two to three days for six months until drug resistance is developed (manuscript under preparation). The cisplatin-resistant cell lines (HK1-LMP1 CisR and HONE1-EBV CisR) and their parental cisplatin-non-resistant cell lines (HONE1-EBV and HK1-LMP1) were provided by Professor Brigette Ma & Dr. Eric Wong from the Department of Clinical Oncology, Chinese University of Hong Kong. The two immortalized normal lung cell lines, namely, HBE and 16HBEo- was obtained from Professor Wen Chen from the Faculty of Preventive Medicine, School of Public Health, Sun Yat-Sen University and Professor Dieter Gruenert from the University of California, San Francisco School of Medicine. The intestinal cancer cell line (CaCo-2) and immortalized intestinal normal cell line (FHs74Int) and the whole series of lung cancer cell lines (A549, NCI-H520, HCC827, NCI-H3255, HCC2935, NCI-H358, NCI-H1650, NCI-H23, NCI-H1975 & NCI-H820) with and without resistance to the anti-EGFR targeted drug, Erlotinib were

acquired from ATCC.

Supplementary Table 1- Lists out the histology and reference of all cell lines used in this study. For the drug resistivity of lung series used, the corresponding IC50 values can be found in reference [S1, 2]

	Cell lines	Cell line histology & characteristics	References
Nasopharyngeal	HONE1	Poorly differentiated squamous carcinoma cell line	S3
	CNE2	Poorly differentiated squamous carcinoma cell line	S4, 5
	HK1	Well differentiated squamous carcinoma cell line	S6
	NP69	Immortalized nasopharyngeal-derived epithelial cell line	S7
	NP460	Immortalized nasopharyngeal-derived epithelial cell line	S8
	HONE1-EBV	Poorly differentiated squamous carcinoma cell line transfected with EBV	S3, 9
	HK1-LMP1	Differentiated squamous carcinoma cell line transfected with LMP1	S6, 10
	HONE1-AUY R	Poorly differentiated squamous carcinoma cell line with AUY922 resistance	(Yip TTC et al., unpublished data)
	HONE1-EBV (Cis	Poorly differentiated squamous	S3, 9, 10

	R)	carcinoma cell line transfected with EBV & with cisplatin resistance	
	HK1-LMP1 (CisR)	Well differentiated squamous carcinoma cell line transfected with LMP1 & with cisplatin resistance	S6, 10
Lung	A549 *	Lung adenocarcinoma cell line	S1, 11
	NCI-H520 *	Lung squamous cell carcinoma cell line	S1, 12
	16HBEo-	Bronchial epithelial cell line	S13
	HBE	Bronchial epithelial cell line transfected with H-Ras	S13
	HCC827 *	Lung adenocarcinoma cell line	S1, 14
	NCI-H3255 *	Lung adenocarcinoma cell line	S1, 15
	HCC2935 *	Lung adenocarcinoma cell line	S1, 16
	NCI-H358 *	Lung bronchioalveolar carcinoma cell line	S1, 17
	NCI-H1650 *	Lung bronchoalveolar carcinoma cell line	S1, 18
	NCI-H23 *	Lung adenocarcinoma cell line	S1, 19
	NCI-H1975 *	Lung adenocarcinoma cell line	S1, 18
	NCI-H820 *	Lung adenocarcinoma cell line	S1, 20

Colorectal	Caco2	Colorectal adenocarcinoma cell line	S21
	FHs74Int	Small intestine epithelial cell line	S22

* IC50 references values can be found in the cited paper [S1, 2]

HONE1, HK1 and NCI-H520 were cultured in RPMI-1640 medium (Gibco) supplemented with 10% fetal bovine serum (FBS) (Sigma) and 1% antibiotic solution containing penicillin & streptomycin (P/S solution, Sigma). CNE2 was cultured in DMEM medium with high glucose (Gibco) supplemented with 5% FBS (Sigma), 5% Newton Calf Serum (Gibco) and 1% P/S solution (Sigma). NP69 was cultured in Keratinocyte-SFM medium (Gibco) supplemented with human recombinant Epidermal Growth Facter 1-53 (Gibco) and Bovine Pituitary extract (Gibco). NP460 was cultured in a 1:1 ratio of Defined Keratinocyte-SFM (Gibco) and EpiLife medium (Gibco) supplemented with EpiLife Defined Growth Supplement – EDGS (Gibco). A549 was cultured in DMEM Glutamax® medium (Gibco) supplemented with 10% FBS (Sigma) and 1% P/S solution (Sigma). HBE was cultured in Minimum Essential Medium (MEM), supplemented with 10% FBS. 16HBE14o- was cultured in MEM supplemented with 0.292g/L L-Glutamine, 1g/L Glucose and 2.2g/L $NaHCO_3$ and seeded on fibronectin coated culture dishes, prepared by immersing the dish in a solution composed of 88% LHC basal medium (Invitrogen), 10% Bovine serum albumin (diluted to 0.1mg/mL, Sigma), 1% of Vitrogen 100 (3mg/mL BD-Laboratories) and 1% Human fibronectin (1mg/mL, BD-Laboratories). Caco-2 was cultured in Eagle’s minimal essential medium supplemented with 20% FBS (Sigma). FHs74Int was cultured in Hybrid-care medium (ATCC) supplemented with 30ng/mL Epidermal growth factor (Sigma).

Cells were maintained at 37°C and 5% CO_2 humidified atmosphere. In addition, a total concentration of 2×10^5 cells/mL was incubated in confocal dishes without serum/growth factor for 24 hours to achieve G0 phase synchronization prior to the actual test.

C. Drugs and protocols for developing drug-resistance

Supply of HSP-90 inhibitor drug, AUY922 was obtained from Novartis Pharmaceuticals (HK) Ltd. The NPC sub-line (HONE1-AUY922-R) which is resistant to AUY922 was established by pulsing HONE1 cell line with 30 nM AUY922 repeatedly every two to three days for six months until drug resistance is developed. The cisplatin-resistant cell lines (HK1-LMP1 CisR and HONE1-EBV CisR) were provided by Professor Brigette Ma & Dr. Eric Wong from the Department of Clinical Oncology, Chinese University of Hong Kong. The whole series of lung cancer cell lines with and without resistance to the anti-EGFR targeted drug, Erlotinib were acquired from ATCC. For other details of cell lines and drug resistance nature, please refer to Supplementary Table 1.

D. Cortical tension measurement

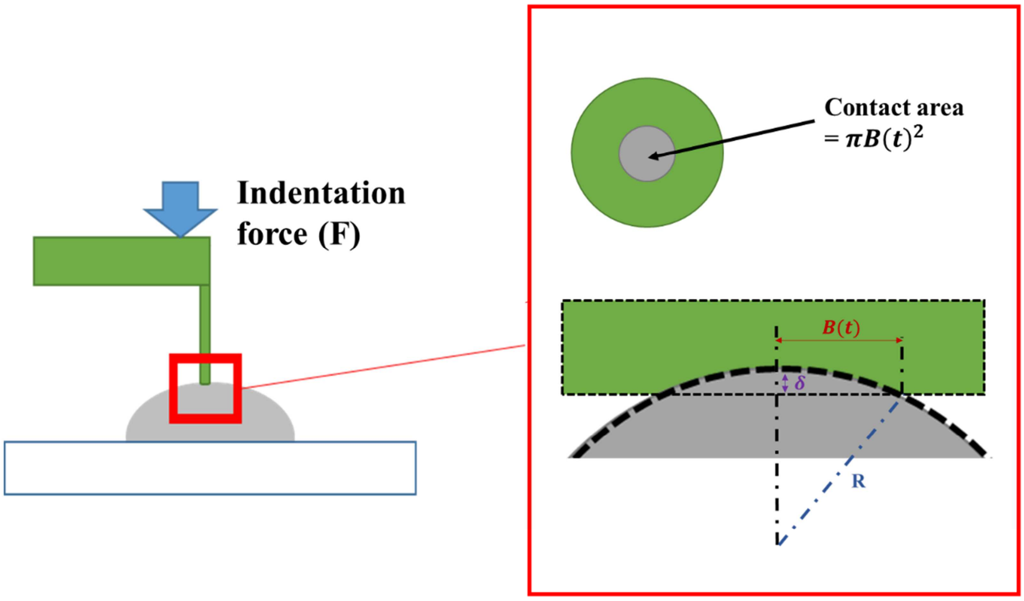

Supplementary Figure 3 Cortical tension measurement via nanoindentation with a flat-end cylindrical punch.

The same protocol used in the mechanical puncture tests (Supplementary Information A), without the retraction stage at the end, was adopted here. In particular, the contact force between the cell and the cylindrical probe during the indenting and subsequent holding stages was recorded which, in conjunction with a simple model, enabled the so-called cortical tension of cells to be estimated. In our experiments, each cell was indented five times at different locations.

Recent studies have shown that the cortical tension of cells can be measured by indentation with a pyramid or spherical indenter [11, S23]. Similar approach was adopted in this investigation. In particular, a cylindrical probe coated with Bovine Serum Albumin

(to eliminate possible adhesion with the cell membrane) was used to indent the cell. Before any contact take place, Laplace law implies that:

$$P_c - P_0 = \frac{2\gamma_m}{R} \tag{S1}$$

where P_c is the intracellular pressure, P_0 is the external pressure, γ_m is the membrane tension and with R is the radius of the cell. Denoting the indentation depth and the corresponding contact radius as δ and B respectively (Supplementary Fig. 3), we proceed by assuming that the cell deformation is small (i.e. the cell still maintains the spherical shape outside the contact region) and hence these two quantities can be approximately related to each other as:

$$B = \sqrt{\delta[2R - \delta]}. \tag{S2}$$

In addition, force equilibrium of the contact part requires that:

$$P_c - P_0 = \frac{F}{\pi B^2} \tag{S3}$$

where F is the force applied by the indenter. Combining Eqs. (S1) – (S3), we finally have:

$$\gamma_m = \frac{FR}{2\pi\delta[2R-\delta]}. \tag{S4}$$

Given that both F and δ are recorded in the indentation test, Eq. (S4) provides a simple way for us to estimate the cortical tension (γ_m). In our experiments, this quantity was measured to be in the range of ~50-200 $\mathrm{pN/\mu m}$ (see Fig. 4(A) and 4(B)), depending on

the cell types, which is comparable to those reported in the literature [S23, 24].

Supplementary Table 2 - List of parameters used in this study

**Parameter**	**Sym**	**Adopted Value**	**Reference Value**	**Source**
Line tension	σ	$1.8 \times 10^{-11} \mathrm{Jm}^{-1}$	$1.8 \times 10^{-11} Jm^{-1}$	S25-27
Diffusion coefficient	D	$1.5 \times 10^{-18}\ \mathrm{m^2 s^{-1}}$	$10^{-16} - 10^{-20}\ \mathrm{m^2 s^{-1}}$	S28-30
Initial pore radius	r_0	0.5 μm	0.5 μm	Controlled in Experiment
Initial indentation depth	δ	0.4 μm		Controlled in Experiment
Cell radius	R	10 μm		Estimated from observation

E. Resealing response of drug-treated A549 cells

To confirm whether the distinct resealing response observed in this study is caused by the different tension levels in the membrane of cells. We treated A549 cells with three drugs, Jasplakinolide (Jas), Cytochalasin D (CytD) and Blebbistain (Blebst), that are known to alter the cortical tension in cells. Specifically, Jas can interfere with actin assembly/disassembly in the cortex and lead to a reduction in the membrane tension by more ~80% on average [S31, 32]; CytD promotes the depolymerization of F-actin and hence will also result in a decrease in the cortical tension [S33, 34]; Blebbistatin inhibits the

contractility of myosins and hence reduces the tension level in cells [25]. Indeed, our measurements show that the tension level in A549 cells treated with Jas (1.5μM for 30 minutes), CytD (0.5μM for 30 minutes) and Blebst (75μM for 30 minutes) was significantly reduced (Fig. 4C). Interestingly, as expected, such decrease in the membrane tension was accompanied by a marked increase in the resealing speed, refer to Fig. 4D. All groups were found to be significantly different from one another (two-sampled t-tests, $P < 0.05$)

F. Resealing response of A549 cells under elevated surrounding osmotic pressure

In addition to drug treatment, variations in the surrounding osmotic pressure (30, 50, and 70mmHg) were also introduced to A549 cells for 2 hours before membrane resealing test was conducted. Specifically, salt solutions were prepared by dissolving sucrose (IBI Scientific) in DMEM medium (Gibco) with 10% (v/v) fetal bovine serum and 1% (v/v) antimycotic as well as antibiotic solution (Invitrogen). The osmotic pressure $\Delta\pi$ can be calculated by the Morse equation

$$\Delta\pi = ick_B T \tag{S5}$$

where i is the dimensionless Van't Hoff factor, c is the concentration of newly added sucrose in the culture medium, k_B is the Boltzmann constant and T is the absolute temperature. Note that the elevated surrounding osmolarity will induce the shrinkage of cells and hence reduce their membrane tensions. As such, it is conceivable that a faster resealing response will be observed here (similar to that shown in Fig. 4). Indeed, as demonstrated in Supplementary Figure 4, a higher increase in the medium

osmolarity will lead to a shorter membrane resealing time.

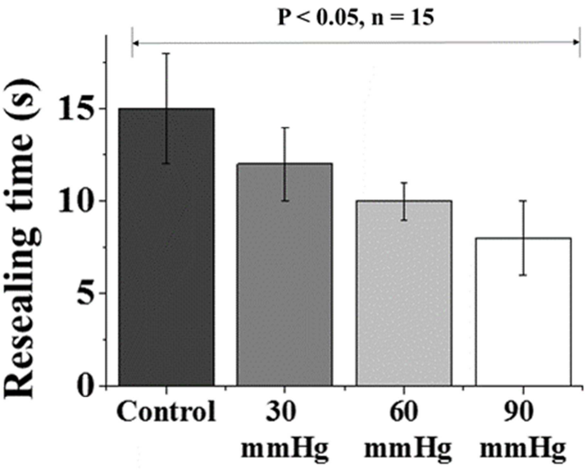

Supplementary Figure 4 The resealing time of 1-μm membrane pores in A549 cells under elevated surrounding osmotic pressure. Results shown here were based on measurements on 15 cells (in each case) and a statistical confidence level of no less than 95% by t-test has been achieved.

G. Effective elastic moduli of cancer and normal cells

The effective elastic moduli of different cancer and normal cells were measured by rate-jump indentation [S35]. Specifically, the synchronized cells were cultured onto the petri dishes for 24 hours. After that, the indenter was first moved into the cell until an indentation depth of 0.2μm is reached, and then was held for 30s, finally followed by a sudden retraction at a speed of 0.1 μm/s for 10s. All atomic force microscopy measurements were obtained by a JPK NanoWizard® II AFM (Veeco, Santa Barbara, CA) that was mounted onto a Zeiss AxioObserver with the fluid-cell-mounted cantilever (Microlevers, Veeco). The piezo platform and photodiode signal were controlled by JPK NanoWizard® II software (JPK Instrumental). A flat-end cylindrical

silicon nitride cantilever (Veeco) of $1\mu m$ in diameter with a spring constant of 0.035 N/m was used in all experiments. All tests were conducted at $25°C$ and within 1 hour after cells were removed from the incubator.

A typical force curve from such test is shown in Supplementary Figure 5A. From which the reduced modulus E_r of the cells can be determined as [S35, 36]

$$\Delta\dot{F} = 2E_r a\Delta\dot{\delta} \tag{S6}$$

where $\Delta\dot{F}$ is the loading rate jump, $\Delta\dot{\delta}$ is the displacement rate jump, and a is the tip radius. Notice that, here $\frac{1}{E_r} = \left[\frac{1-\nu^2}{E}\right]_{tip} + \left[\frac{1-\nu^2}{E}\right]_{cell}$ with E and ν being the Young's modulus and the Poisson's ratio respectively. The indenter itself is much stiffer than the cell, and hence the Young's modulus of the cell, i.e. E_{cell}, can be estimated from E_r, measured from eqn. (S6), as

$$E_{cell} = \frac{3}{4}E_r \tag{S7}$$

where it has been assumed that $\nu_{cell} \approx 0.5$ (i.e. the cell body is treated as incompressible). By conducting indentation on 15 different synchronized cells with fragmented cells rejected, the average cell modulus was shown in Supplementary Figure 5B. Clearly, tumor cells all appear to be softer than the corresponding normal ones.

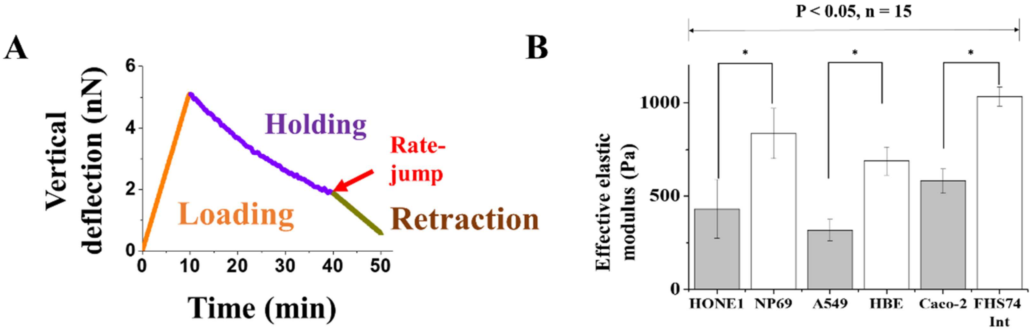

Supplementary Figure 5 **A -** A typical force curve obtained from the rate jump indentation test on HONE1 cells. **B –** Effective elastic moduli of different cancer (HONE1, A549 and Caco-2) cells and their normal counterparts (i.e. NP69, HBE and FHS74Int cells).

References

S1. Sos, M. L. et al. *Cancer Res* **2009,** 69, (8), 3256-61.

S2. Furugaki, K. et al. *Oncol Rep* **2010,** 24, (5), 1141-6.

S3. Yao, K. T. et al. *Int J Cancer* **1990,** 45, (1), 83-9.

S4. Lerman, M. I.; Sakai, A.; Yao, K. T.; Colburn, N. H. *Carcinogenesis* **1987,** 8, (1), 121-7.

S5. Sizhong, Z.; Xiukung, G.; Yi, Z. *Int J Cancer* **1983,** 31, (5), 587-90.

S6. Huang, D. P. et al. *Int J Cancer* **1980,** 26, (2), 127-32.

S7. Zhang, H. et al. *Cancer genetics and cytogenetics* **2004,** 150, (2), 144-52.

S8. Li, H. M. et al. *International journal of cancer. Journal international du cancer* **2006,** 119, (7), 1567-76.

S9. Chen, J. et al. *J Transl Med* **2010,** 8, 30.

S10. Ma, B. B. et al. *Invest New Drugs* **2010,** 28, (4), 413-20.

S11. Lieber, M. et al. *International journal of cancer. Journal international du cancer* **1976,** 17, (1), 62-70.

S12. Banks-Schlegel, S. P.; Gazdar, A. F.; Harris, C. C. *Cancer Res* **1985,** 45, (3), 1187-97.

S13. Tsao, M. S.; Zhu, H.; Viallet, J. *Exp Cell Res* **1996,** 223, (2), 268-73.

S14. Girard, L. et al. *Cancer Res* **2000,** 60, (17), 4894-906.

S15. Fujishita, T. et al. *Oncology* **2003,** 64, (4), 399-406.

S16. Varella-Garcia, M. et al. *Cancer research* **2005,** 65, (9 Supplement), 393.

S17. Brower, M. et al. *Cancer Res* **1986,** 46, (2), 798-806.

S18. Phelps, R. M. et al. *J Cell Biochem Suppl* **1996,** 24, 32-91.

S19. Gazdar, A. F. et al. *Cancer research* **1980,** 40, (10), 3502-7.

S20. Takahashi, T. et al. *Science* **1989,** 246, (4929), 491-4.

S21. Fogh, J.; Wright, W. C.; Loveless, J. D. *Journal of the National Cancer Institute* **1977,** 58, (2), 209-14.

S22. Owens, R. B.; Smith, H. S.; Nelson-Rees, W. A.; Springer, E. L. *J Natl Cancer Inst* **1976,** 56, (4), 843-9.

S23. Lomakina, E. B.; Spillmann, C. M.; King, M. R.; Waugh, R. E. *Biophys J* **2004,** 87,

(6), 4246-58.

S24. Hochmuth, R. M. *J Biomech* **2000,** 33, (1), 15-22.

S25. Kroeger, J. H.; Vernon, D.; Grant, M. *Biophys J* **2009,** 96, (3), 907-16.

S26. Freeman, S. A.; Wang, M. A.; Weaver, J. C. *Biophys J* **1994,** 67, (1), 42-56.

S27. Glaser, R. W. et al. *Biochim Biophys Acta* **1988,** 940, (2), 275-87.

S28. Derzko, Z.; Jacobson, K. *Biochemistry* **1980,** 19, (26), 6050-6057.

S29. Schneider, M. B.; Chan, W. K.; Webb, W. W. *Biophys J* **1983,** 43, (2), 157-65.

S30. Jacobson, K. *Cell Motility* **1983,** 3, (5), 367-373.

S31. Lieber, A. D. et al. *Current Biology* **2013,** 23, (15), 1409-1417.

S32. Boulant, S. et al. *Nat Cell Biol* **2011,** 13, (9), 1124-1131.

S33. Togo, T.; Krasieva, T. B.; Steinhardt, R. A. *Mol Biol Cell* **2000,** 11, (12), 4339-46.

S34. Ting-Beall, H. P.; Lee, A.; Hochmuth, R. *Annals of Biomedical Engineering* **1995,** 23, (5), 666-671.

S35. Zhou, Z. L.; Ngan, A.H.W.; Tang, B.; Wang A.X. *Journal of the Mechanical Behavior of Biomedical Materials*, **2012**, 8, 134-142.

S36. Tang, B.; Ngan, A.H.W. *Soft Matter*, **2012**, 8, 5974-5979.
